# Supplementary material for: A UMLS-based spell checker for natural language processing in vaccine safety
Source: BMC Med Inform Decis Mak. 2007 Feb 12;7:3. doi: 10.1186/1472-6947-7-3 (PMC1805499; doi:10.1186/1472-6947-7-3)
Supplement: Additional file 13 — RAP application directory. Sets up directory for RAP (RDF application for PHP) source code [file 1472-6947-7-3-S13.gz › rap/doc/phpdoc/rdql/RdqlResultIterator.html]

xml version="1.0" encoding="iso-8859-1"?


Docs For Class RdqlResultIterator


## Class RdqlResultIterator

Description

Description |
Methods (details)

Iterator for traversing Rdql results.

This class can be used for iterating forward and backward trough Rdql results. It should be instanced using the rdqlQueryAsIterator() method of a MemModel or a DBModel.

History:

- 25-03-2004 : Bug in the handling of empty results fixed (radol@gmx.de).
- 11-06-2003 : First version of this class.

- access: public
- author: Daniel Westphal <mail@d-westphal.de>, Chris Bizer <chris@bizer.de>
- version: V0.9.1

Located in /rdql/RdqlResultIterator.php (line 25)

```
Object
   |
   --RdqlResultIterator
```

Method Summary

Description |
Methods (details)

RdqlResultIterator
RdqlResultIterator
(object RdqlResult &$RdqlResult)

integer
countResults
()

result
current
([integer $element = null])

integer
getCurrentPosition
()

array
getResultLabels
()

boolean
hasNext
()

boolean
hasPrevious
()

void
moveFirst
()

void
moveLast
()

void
moveTo
(mixed $position)

result
next
([integer $element = null])

result
previous
([integer $element = null])

Methods

Description |
Methods (details)

Constructor RdqlResultIterator (line 50)

Constructor

- access: public

RdqlResultIterator

RdqlResultIterator
(object RdqlResult &$RdqlResult)

- object RdqlResult
  &$RdqlResult

countResults (line 83)

Returns the number of results.

- access: public

integer

countResults
()

current (line 151)

Returns the current result.

- return: array OR single result if $element was specified OR NULL if there is no next result.
- access: public

result

current
([integer $element = null])

- integer
  $element

getCurrentPosition (line 193)

Returns the current position of the iterator.

- access: public

integer

getCurrentPosition
()

getResultLabels (line 72)

Returns the labels of the result as array.

- return: of strings with the result labels OR null if there are no results.
- access: public

array

getResultLabels
()

hasNext (line 93)

Returns TRUE if there are more results.

- access: public

boolean

hasNext
()

hasPrevious (line 106)

Returns TRUE if the first result has not been reached.

- access: public

boolean

hasPrevious
()

moveFirst (line 165)

Moves the pointer to the first result.

- access: public

void

moveFirst
()

moveLast (line 174)

Moves the pointer to the last result.

- access: public

void

moveLast
()

moveTo (line 184)

Moves the pointer to a specific result.

If you set an off-bounds value, next(), previous() and current() will return NULL

- access: public

void

moveTo
(mixed $position)

next (line 119)

Returns the next result array.

- return: array OR single result if $element was specified OR NULL if there is no next result.
- access: public

result

next
([integer $element = null])

- integer
  $element

previous (line 135)

Returns the previous result.

- return: array OR single result if $element was specified OR NULL if there is no next result.
- access: public

result

previous
([integer $element = null])

- integer
  $element

#### Inherited Methods


Inherited From Object

> Object::toString()

Documentation generated on Thu, 7 Jul 2005 13:43:28 +0200 by phpDocumentor 1.3.0RC3
